# Exoskeleton therapy in cerebral palsy: improved gait endurance without kinematic change

**DOI:** 10.3389/fnhum.2025.1644585

**Published:** 2025-08-07

**Authors:** Paweł Chmara, Sabina Brazevic, Marek Jóźwiak, Brian Po-Jung Chen, Faustyna Manikowska

**Affiliations:** ^1^Gait and Motion Analysis Laboratory, Department of Pediatric Orthopedics and Traumatology, Poznań University of Medical Sciences, Poznań, Poland; ^2^Motion Analysis Laboratory, Bone and Joint Research Center, Chang Gung Memorial Hospital - Linkou, Taoyuan, Taiwan

**Keywords:** robotic-assisted gait training, exoskeleton, cerebral palsy, six-minute walk test, gait

## Abstract

**Introduction:**

Cerebral palsy (CP) often leads to impairments in movement and posture, limiting functional mobility. Robotic-assisted gait training (RAGT) using powered exoskeletons has emerged as a novel approach to enhance gait in individuals with CP. However, evidence regarding its effectiveness, particularly in unassisted gait performance, remains limited and inconclusive.

**Methods:**

This study involved 44 ambulatory youth with bilateral hypertonic CP (GMFCS levels I–III), who underwent an intensive RAGT program using the EksoGT exoskeleton. The intervention consisted of 28 sessions (90 min each) over 8 weeks, with a 2 weeks mid-point break. Gait assessments were conducted before (T1) and after (T2) therapy using 3D motion analysis and the 6-minute walk test (6MWT). Primary outcomes included spatiotemporal parameters, gait symmetry, gait deviation index (GDI), and walking endurance.

**Results:**

Following the exoskeleton training, participants demonstrated a statistically significant improvement in gait efficiency, with 6MWT distances increasing from 375 to 418 m (*p* < 0.01). However, no significant changes were observed in gait symmetry, spatiotemporal parameters, or GDI scores, indicating no measurable effect on unassisted gait mechanics.

**Conclusion:**

Intensive exoskeleton therapy significantly improved walking endurance but did not alter gait symmetry or kinematics in independently ambulatory youth with hypertonic CP. These - findings suggest that while exoskeleton assisted training enhances functional endurance, its impact on gait pattern may be limited. Further research should explore its broader benefits on quality of life, participation, and psychosocial outcomes.

## 1 Introduction

Children with cerebral palsy (CP), resulting from a non-progressive brain lesion, present with permanent disorders of movement and posture that lead to functional disability (Rosenbaum et al., 2007). The combination of multiple impairments contributes to abnormal muscle activity and movement patterns, which can be categorized as either positive motor signs (e.g., hypertonia and involuntary movements) or negative motor signs (e.g., muscle weakness and impaired control) (Sanger et al., 2006). These motor deficits collectively limit functional performance.

Among the available interventions, task-specific functional therapy has been identified as the most effective approach for enhancing gross motor function in children with CP. This method emphasizes active participation in daily life activities that involve purposeful movement execution to improve coordination and motor performance (Christy et al., 2012; Graham et al., 2016). Active engagement, both physical and cognitive, is essential for therapeutic success. In contrast, passive interventions such as manual manipulation by a therapist or mechanical assistance using devices like exoskeletons have shown limited efficacy, potentially due to reduced engagement of both the patient and their nervous system (Graham et al., 2016).

Robotic-assisted gait training (RAGT) has emerged as a widely used rehabilitation modality to improve gait in individuals with neurological impairments. Various forms of RAGT, including driven gait orthoses and powered lower limb exoskeletons, aim to enhance voluntary movement control by adapting joint motion (e.g., at the ankle or knee), measuring human-machine interaction forces, supporting leg movement, providing body weight support, and maintaining gait trajectories. These systems have demonstrated benefits in children with CP (Banz et al., 2008; Brütsch et al., 2010; Jamwal et al., 2020). However, despite promising results, the overall evidence remains limited due to the lack of standardized outcome measures. Furthermore, the comparative efficacy of RAGT versus conventional therapy remains under debate (Bunge et al., 2021; Cortés-Pérez et al., 2022).

According to the World Health Organization’s International Classification of Functioning, Disability and Health (ICF), promoting independence in daily activities is a key therapeutic goal (Gormley, 2001; Gómez-Salgado et al., 2018). While powered exoskeletons, one of the most widely used RAGT devices, involve some degree of passive assistance, they enable overground walking that closely resembles natural daily activity and require less hands-on input from the therapist. This allows for more cognitively engaging practice, potentially enhancing therapeutic outcomes (Fasoli et al., 2012).

The aim of this study was to evaluate the effect of powered lower limb exoskeleton use on gait parameters in children with spastic CP. Specifically, the study investigated short-term changes in spatiotemporal gait parameters, gait kinematics, and gait asymmetry.

## 2 Materials and methods

### 2.1 Participants

A total of 44 subjects diagnosed with hypertonic CP were recruited for the study and underwent exoskeleton-assisted gait training (20 females, 24 males; mean age: 17.61 ± 3.95 years). The study group consisted of ambulatory individuals with bilateral lower limb involvement. All participants were able to walk independently or with assistive devices and were classified within levels I to III of the Gross Motor Function Classification System (GMFCS): Level I (*n* = 5), Level II (*n* = 31), and Level III (*n* = 8).

Inclusion criteria were as follows: (1) diagnosis of bilateral hypertonic CP, (2) no orthopedic surgery within the past year, (3) no botulinum toxin injections within the last 6 months, (4) ability to follow verbal instructions, and (5) a primary therapeutic goal of improving gait function. Exclusion criteria included: (1) the presence of pain, (2) fixed contractures that prevented participation in training, and (3) leg length discrepancy greater than 0.5 cm.

Gait training sessions were conducted at the outpatient clinic of a local rehabilitation hospital. All assessments were performed at the Motion Analysis Laboratory of a local orthopedic hospital. The study was approved by the appropriate Institutional Review Board. Written informed consent was obtained from all participants aged 18 years or older and from parents or legal guardians for those under 18 years of age.

### 2.2 Protocol

Each participant underwent a total of 28 therapy sessions of exoskeleton training over a period of 8 consecutive weeks, with a 2 weeks break at the midpoint. Each session lasted 90 min and included the following components:

1. Warm-up: Strength and balance exercises to prepare for gait training.
2. Therapy session: 40 min of walking practice using the exoskeleton device.
3. Cool-down: Stretching exercises to conclude the session.

Exoskeleton training was conducted using the EksoGT powered exoskeleton (Ekso Bionics Holdings, Inc., San Rafael, CA, United States). The device is designed for users with a body weight of up to 100 kg, a height range of 1.58–1.88 m, and a maximum hip width of 45.7 cm. The system weighs 27 kg and includes two lithium-ion batteries (2 Ah, 48.1 VDC) capable of producing a peak current of 30 A, allowing full-load operation for up to 60 min.

The EksoGT supports active hip flexion from −20° to 135° and passive hip abduction from −2° to 4°. Knee flexion is motor-assisted within a 0°–135° range, while the ankle allows passive plantarflexion from −10° to 10°. Foot stiffness can be adjusted on a scale of 1 (flexible) to 4 (rigid). The device enables a walking speed of approximately 2 km/h, with adjustable step lengths (20.3–45.7 cm), step widths (0.0–7.6 cm), and swing phase durations (0.8–2.5 s). Before the first therapy session, each participant received a 30–60 min adaptation period to become familiar with walking in the exoskeleton.

Gait performance was assessed twice for each subject: prior to the intervention (T1) and immediately after completing the 28 therapy sessions (T2). Assessments included instrumental gait analysis and the six-minute walk test (6MWT) (Enright, 2003). The 6MWT was used to evaluate gait endurance. During the test, participants walked at their maximum achievable speed along a 15 m straight course for 6 min. The course was marked on a flat surface using cones, with each meter clearly labeled. An examiner recorded the distance covered using a stopwatch, and the total distance was calculated by summing the number of completed laps and any additional meters from the final, incomplete lap.

Kinematic data were collected using an 8-camera motion capture system (six Bonita 3 and two Vero 2.2; Vicon Motion Systems Ltd., Oxford, United Kingdom) at a sampling rate of 120 Hz. Reflective markers were placed according to the standard Lower Body Plug-in-Gait protocol. All participants walked barefoot along a 10 m walkway at a self-selected speed during data collection.

### 2.3 Outcome measure

The primary outcome measures included the following gait spatiotemporal parameters: step time (s), step length (m), step width (m), stride time (s), stride length (m), walking speed (m/s), cadence (steps/min), single support (% of gait cycle), double support (% of gait cycle), and foot-off (% of gait cycle). In addition, the gait symmetry index (SI) was calculated for each of these parameters. Further outcome measures included the Gait Deviation Index (GDI) and the total distance covered in the 6MWT.

Gait symmetry was calculated based on the spatiotemporal parameters using the following formula: (Robinson et al., 1987)


$$S\,I=\frac{2\,\left(X_{R}-X_{L}\right)}{X_{R}+X_{L}}\times 100\%$$

where:


$$X_{R}:r\,i\,g\,h\,t\,l\,e\,g\,p\,a\,r\,a\,m\,e\,t\,e\,r$$

$$X_{L}:l\,e\,f\,t\,l\,e\,g\,p\,a\,r\,a\,m\,e\,t\,e\,r$$

An SI value of 0 indicates perfect symmetry; negative values indicate asymmetry favoring the left side, while positive values indicate asymmetry favoring the right.

Changes in GDI values were categorized as improvement when ΔGDI ≥ 5, deterioration when ΔGDI ≤ −5, and no change when −5 < ΔGDI < 5 (Schwartz et al., 2016; Rajagopal et al., 2018).

### 2.4 Statistical analysis

The normality of data distribution was assessed using the Shapiro-Wilk test. For variables that were normally distributed and exhibited homogeneity of variance, paired *t*-tests were used to evaluate changes over time. For non-normally distributed variables or those with heterogeneous variance, the Wilcoxon signed-rank test was applied. Differences in the Gait Deviation Index (GDI) were analyzed using the Fisher-Freeman-Halton test. All statistical analyses were conducted using the Statistical software (Version 14; TIBCO Software Inc., Palo Alto, CA, United States). A *p*-value of < 0.05 was considered statistically significant.

## 3 Results

### 3.1 Six-minute walk test (6MWT)

**FIGURE 1:**
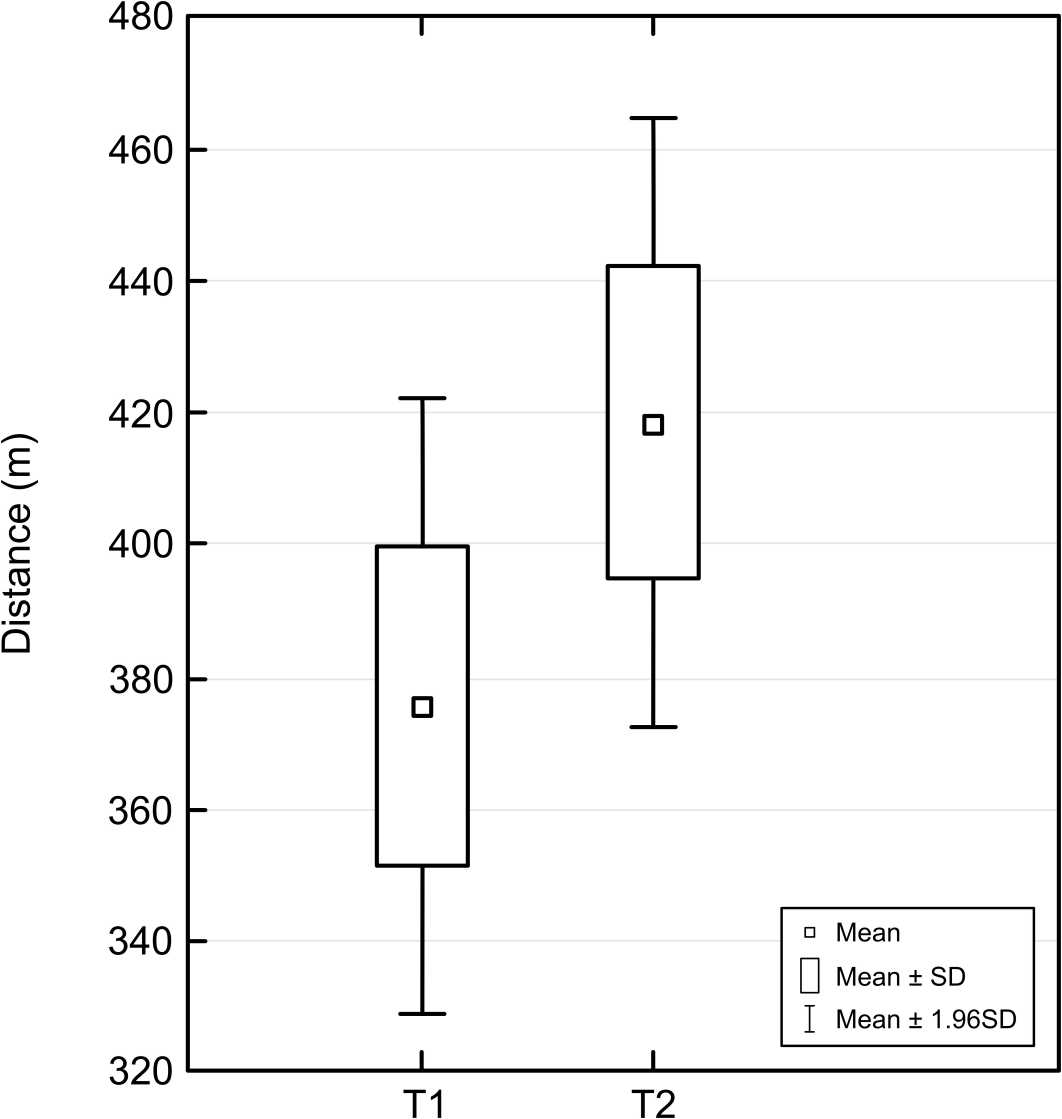
Changes in six-minute walk test (6MWT) distance before (T1) and after (T2) robotic-assisted gait training (RAGT).

Analysis of the effect of exoskeleton therapy on gait efficiency in patients with CP demonstrated a statistically significant improvement in the distance covered during the 6MWT (T1 = 375 m; T2 = 418 m; *p* < 0.01; normally distributed; Figure 1).

### 3.2 Gait symmetry

No statistically significant changes in gait symmetry were observed following exoskeleton therapy (Table 1).

**TABLE 1:** Gait symmetry index before (T1) and after (T2) the exoskeleton therapy.

		T1			T2			*P*-value
	Collected variables	(Mean ± SD)	(Median)	(IQR)	(Mean ± SD)	(Median)	(IQR)	
Symmetry Index (%)	Walking speed	−0.2 ± 2.1	0.0	2.1	−0.2 ± 4.4	0.0	3.7	0.54 0.32 0.97 0.70 1.00 0.73 0.16 0.86 0.68 0.60
	Stride time	0.0 ± 1.3	0.0	1.6	0.4 ± 2.1	0.4	1.8	
	Step time	3.3 ± 14.4	3.4	11.8	4.3 ± 14.6	4.0	13.1	
	Double support*	4.7 ± 23.3	0.6	26.4	3.9 ± 22.6	0.6	26.4	
	Single support	−1.0 ± 11.8	−1.8	13.6	−1.1 ± 11.7	−2.4	11.9	
	Foot off	−0.2 ± 7.2	−0.9	5.2	−0.3 ± 5.7	−0.2	7.2	
	Stride length	−0.1 ± 2.0	0.0	2.3	1.2 ± 8.3	0.4	2.9	
	Step length	2.5 ± 16.3	3.2	13.3	3.5 ± 14.5	4.3	14.3	
	Step width	−1.0 ± 8.5	0.0	0.0	−0.7 ± 9.3	0.0	0.0	
	GDI*	−1.3 ± 15.0	0.3	20.9	−2.2 ± 13.7	−1.9	15.7	

The *p*-value indicates the statistical significance of differences between T1 and T2.

*, normally distributed variables; SD, standard deviation; IQR, interquartile range; GDI, Gait Deviation Index.

### 3.3 Spatiotemporal gait parameters

Analysis of spatiotemporal gait parameters revealed no statistically significant changes after the intervention (Table 2).

**TABLE 2:** Spatiotemporal parameters before (T1) and after (T2) the exoskeleton therapy.

	T1			T2			*P*-value
Collected variables	(Mean ± SD)	(Median)	(IQR)	(Mean ± SD)	(Median)	(IQR)	
Cadence (step/min)	98.89 ± 17.10	102.48	16.49	98.79 ± 15.48	101.77	16.50	0.44
Walking speed* (m/s)	0.79 ± 0.27	0.83	0.33	0.80 ± 0.28	0.80	0.47	0.59
Stride time (s)	1.26 ± 0.28	1.17	0.21	1.26 ± 0.28	1.18	0.25	0.27
Step time (s)	0.60 ± 0.14	0.64	0.13	0.60 ± 0.15	0.55	0.13	0.21
Double support (% of GC)	22.83 ± 12.76	17.98	13.03	22.78 ± 10.97	18.30	13.63	0.59
Single support (% of GC)	43.28 ± 9.33	49.58	13.45	43.78 ± 8.91	49.85	15.15	0.36
Foot off (% of GC)	66.11 ± 5.17	64.58	5.08	66.56 ± 3.90	66.33	4.22	0.18
Stride length* (m)	0.95 ± 0.25	0.96	0.39	0.96 ± 0.28	0.97	0.43	0.34
Step length* (m)	0.45 ± 0.12	0.46	0.19	0.46 ± 0.13	0.45	0.18	0.19
Step width* (m)	0.20 ± 0.06	0.19	0.08	0.20 ± 0.06	0.19	0.07	0.89

The *p*-value indicates the statistical significance of differences between T1 and T2.

*, normally distributed variables; SD, standard deviation; IQR, interquartile range; GDI, Gait Deviation Index.

### 3.4 Gait Deviation Index (GDI)

No statistically significant changes in gait kinematics, as measured by the Gait Deviation Index, were found following exoskeleton therapy (Table 3).

**TABLE 3:** Number of participants showing changes in Gait Deviation Index (GDI).

	T2 vs. T1 [*n* (% of group)]	*P*-value
Improvement	9 (20.45%)	0.30
No changes	28 (63.64%)	
Deterioration	7 (15.91%)	

## 4 Discussions

The aim of this study was to evaluate the effectiveness of intensive exoskeleton-assisted therapy on gait in ambulatory individuals with hypertonic CP. Our findings indicate that, while this form of exoskeleton training significantly improved gait efficiency, as measured by the six-minute walk test (6MWT), it did not result in statistically significant changes in gait symmetry, spatiotemporal parameters, or kinematic patterns.

The literature on the efficacy of exoskeleton therapy in individuals with CP remains limited and highly variable in terms of methodology, participant characteristics, and outcome measures (Bunge et al., 2021; Hunt et al., 2022). Previous studies reviewed in this study have been conducted with heterogeneous samples differing in age, diagnosis, functional level, sample size, and therapy duration. Some findings are based on a single session with a single participant, making it difficult to generalize conclusions. To our knowledge, this study represents one of the largest and most homogeneous cohorts to date. We evaluated 44 youths with bilateral hypertonic CP, all ambulatory without third-party assistance (GMFCS levels I–III), and all received the same long-term, intensive exoskeletal training protocol without additional therapeutic interventions. Gait outcomes were assessed using objective, instrumented measures.

Importantly, our study cohort consisted of adolescents and young adults (mean age: 18 years), a population in which functional development and gait patterns are generally considered stable. This minimizes the confounding influence of natural developmental changes on gait, which is a significant concern in studies involving younger children. Previous research has included participants ranging from 5 to 31 years of age, making it difficult to isolate the effects of exoskeleton due to age-related variability in gait (Sutherland, 1997; Ganley and Powers, 2005).

The evidence regarding the effect of exoskeleton on spatiotemporal gait parameters is inconsistent. Some studies report significant improvements, while others find no measurable changes (Bayón et al., 2016; Lerner et al., 2017a; Mataki et al., 2018; Matsuda et al., 2018; Ueno et al., 2019; Nakagawa et al., 2020; Thurston et al., 2021). Additionally, certain studies evaluated gait parameters while participants were wearing the exoskeleton, which can introduce bias. Improvements in parameters such as walking speed, cadence, and step length have been observed in some of these studies, while others report no change, or even a decline (Robinson et al., 1987; Mileti et al., 2016; Lerner et al., 2017d,2017a,2017c; Orekhov et al., 2020). Improvements in gait symmetry and lower limb kinematics during assisted walking have also been documented. However, it is important to recognize that gait patterns induced by powered exoskeletons may not be retained once the device is removed, thus limiting the interpretation of such immediate effects as true functional gains.

Despite the absence of significant changes in kinematic data following therapy, we conducted a detailed analysis of the direction of change in the Gait Deviation Index (GDI). The results showed that GDI improved in nine subjects, declined in seven, and remained unchanged in the majority (28 subjects). Further analysis indicated that GMFCS level was not a distinguishing factor for improvement or deterioration, among those who improved, six were classified as GMFCS level II and 1 as level III.

In contrast to studies assessing gait while wearing the device, we focused on evaluating unassisted, barefoot gait before and after a full course of therapy (28 sessions). Our results did not reveal any significant changes in spatiotemporal parameters, symmetry, or kinematics, suggesting that 28-session exoskeleton training in 8 weeks may not induce lasting modifications in gait pattern among independently ambulatory youth with CP.

Despite the lack of significant changes in gait mechanics, we observed a meaningful improvement in gait efficiency, as reflected in the increased distance covered during the 6MWT. This suggests a potential training effect resulting from sustained walking with resistance. Given the 27 kg weight of the exoskeleton and the intensity of each session (40 min of active walking), it is plausible that this form of therapy contributed to improved muscular endurance and walking capacity. Prior studies have suggested that the weight of the device could impose a metabolic burden (Rossi et al., 2013; Russell Esposito et al., 2018); however, our findings align with literature indicating that resistance or strength-based training can improve function in individuals with CP and support the notion that exoskeleton use may provide a similar benefit.

Most existing studies, including ours, have focused on ambulatory individuals with CP classified as GMFCS levels I–III (Lerner et al., 2016, 2017d,2017b; 2017a; 2017c; 2018; 2019; Takahashi et al., 2018; Orekhov et al., 2020; Chen et al., 2021; Fang et al., 2022). A limited number of studies have examined the effects of exoskeleton in non-ambulatory individuals, i.e., GMFCS level IV (Smania et al., 2012; Nakagawa et al., 2019, 2020; Ueno et al., 2019). However, interpreting gait-related outcomes in non-ambulatory populations may be problematic, as these individuals are not accustomed to autonomous locomotion. In such cases, the value of exoskeleton therapy may lie more in enhancing participation and quality of life rather than altering gait patterns. While theoretical frameworks suggest that exoskeleton could facilitate community participation and mobility, empirical evidence supporting this claim is currently lacking (Bunge et al., 2021).

### 4.1 Limitations

A key limitation of this study is its exclusive focus on gait-related outcomes. Although gait is a critical component of functional mobility, the potential benefits of exoskeleton therapy may extend beyond biomechanics to include psychosocial domains such as participation, self-efficacy, and quality of life. These aspects were not assessed in the present study and should be a focus for future research to comprehensively evaluate the impact of exoskeleton in individuals with CP.

While the current study demonstrated the benefits of an intensive program, future research may be strengthened by incorporating longitudinal follow-up, control groups, and multidimensional outcome measures to better establish the therapeutic potential of robotic gait interventions.

Regarding the statistical analysis used to interpret our results, although appropriate analyses have been applied, additional methods, such as effect size metrics and predictive modeling, could further provide more comprehensive insights and strengthen the interpretation of the therapy program’s effectiveness.

## 5 Conclusion

This study demonstrates that an intensive exoskeleton program can significantly improve gait efficiency in ambulatory youth with hypertonic CP. Despite this improvement in gait endurance, no statistically significant changes were observed in gait symmetry, spatiotemporal parameters, or kinematic profiles. These findings suggest that while the powered exoskeleton may effectively enhance functional walking capacity, it does not appear to meaningfully alter unassisted gait patterns in the short term. Future research should investigate the broader impact of exoskeleton therapy, including its effects on quality of life, social participation, and psychological wellbeing, to better understand its full therapeutic potential.

## Data Availability

The raw data supporting the conclusions of this article will be made available by the authors, without undue reservation.
